# Opposing Effects of Sirtuins on Neuronal Survival: SIRT1-Mediated Neuroprotection Is Independent of Its Deacetylase Activity

**DOI:** 10.1371/journal.pone.0004090

**Published:** 2008-12-31

**Authors:** Jason A. Pfister, Chi Ma, Brad E. Morrison, Santosh R. D'Mello

**Affiliations:** Department of Molecular and Cell Biology, University of Texas at Dallas, Richardson, Texas, United States of America; University of Auckland, New Zealand

## Abstract

**Background:**

Growing evidence suggests that sirtuins, a family of seven distinct NAD-dependent enzymes, are involved in the regulation of neuronal survival. Indeed, SIRT1 has been reported to protect against neuronal death, while SIRT2 promotes neurodegeneration. The effect of SIRTs 3–7 on the regulation of neuronal survival, if any, has yet to be reported.

**Methodology and Principal Findings:**

We examined the effect of expressing each of the seven SIRT proteins in healthy cerebellar granule neurons (CGNs) or in neurons induced to die by low potassium (LK) treatment. We report that SIRT1 protects neurons from LK-induced apoptosis, while SIRT2, SIRT3 and SIRT6 induce apoptosis in otherwise healthy neurons. SIRT5 is generally localized to both the nucleus and cytoplasm of CGNs and exerts a protective effect. In a subset of neurons, however, SIRT5 localizes to the mitochondria and in this case it promotes neuronal death. Interestingly, the protective effect of SIRT1 in neurons is not reduced by treatments with nicotinamide or sirtinol, two pharmacological inhibitors of SIRT1. Neuroprotection was also observed with two separate mutant forms of SIRT1, H363Y and H355A, both of which lack deacetylase activity. Furthermore, LK-induced neuronal death was not prevented by resveratrol, a pharmacological activator of SIRT1, at concentrations at which it activates SIRT1. We extended our analysis to HT-22 neuroblastoma cells which can be induced to die by homocysteic acid treatment. While the effects of most of the SIRT proteins were similar to that observed in CGNs, SIRT6 was modestly protective against homocysteic acid toxicity in HT-22 cells. SIRT5 was generally localized in the mitochondria of HT-22 cells and was apoptotic.

**Conclusions/Significance:**

Overall, our study makes three contributions - (a) it represents the first analysis of SIRT3–7 in the regulation of neuronal survival, (b) it shows that neuroprotection by SIRT1 can be mediated by a novel, non-catalytic mechanism, and (c) that subcellular localization may be an important determinant in the effect of SIRT5 on neuronal viability.

## Introduction

Sirtuins are a family of NAD-dependent enzymes homologous to the yeast Sir2 protein. Overexpression of Sir2 in yeast, *Caenorhabditis elegans* and *Drosophila* increases lifespan by a process believed to be analogous to caloric restriction. More recent work has implicated sirtuins in the control of a variety of biological processes including transcriptional silencing, chromosomal stability, cell cycle progression, apoptosis, autophagy, metabolism, growth suppression, inflammation, and stress response (for recent reviews, [1]–[4]). Mammals express seven sirtuins, termed SIRT1–SIRT7, which are also referred to as class III histone deacetylases (HDACs). The seven enzymes share a conserved catalytic core domain of approximately 275 amino acids but differ in their amino and carboxyl terminal protein sequences flanking this core. Moreover, while SIRT1, SIRT2, SIRT3, and SIRT5 deacetylate histone and non-histone protein substrates, SIRT4 and SIRT6 are primarily mono-ADP-ribosyl transferases [5]–[7]. An activity for SIRT7 has yet to be firmly established. The sirtuins also show differences in their subcellular localization. SIRT1, which has highest sequence similarity to yeast Sir2, is largely nuclear where it deacetylates histones H3 and H4 as well as transcription factors such as NF-κB, p53, FOXO, Ku70, and PGC-1α (reviewed in [1]; [8]). Although generally described to be a nuclear protein, a few recent studies have described nucleo-cytoplasmic shuttling of SIRT1 in response to oxidative stress [8]–[10]. More recently novel co-activators of SIRT1 such as AROS and HIC1 and a co-repressor, DBC1, have also been identified that promote and inhibit SIRT1-mediated deacetylation of its targets [11]–[14]. SIRT2 resides mostly in the cytoplasm where it associates with microtubules and deacetylates α -tubulin [5], [15]. When the nuclear envelope disassembles during mitosis, however, SIRT2 can also deacetylate histone H4 [16]. SIRT3, SIRT4, and SIRT5 localize to the mitochondria and are therefore thought to play a role in energy metabolism and responses to oxidative stress [17]. Within the mitochondria, these SIRTs appear to localize to different sub-compartments, suggesting distinct functions [18]. Like SIRT1, SIRT6 and SIRT7 are nuclear proteins although the three proteins display distinct sub-nuclear localization patterns; SIRT6 associates with heterochromatin, SIRT7 localizes to nucleoli, whereas SIRT1 is largely associated with euchromatin within the nucleus [17].

A growing body of evidence implicates SIRT1 and SIRT2 as important regulators of neurodegeneration [4], [19]. For example, the overexpression of SIRT1 prevents neuronal death in tissue culture models of Alzheimer's disease, amyotropic lateral sclerosis, and polyglutamine toxicity and reduces hippocampal degeneration in a mouse model of Alzheimer's disease toxicity [14], [20]. Moreover, treatment with resveratrol, a polyphenolic compound frequently used as a pharmacological activator of SIRT1, is protective in a variety of experimental paradigms of neurodegeneration (reviewed in [21]). Activation of SIRT1 using other non-commercially available compounds has also been reported to be neuroprotective in a model of optic neuritis [22]. On the other hand, SIRT2 has been reported to promote neuronal death. Pharmacological and genetic inhibition of SIRT2 protects neurons against α-synuclein toxicity *in vitro* as well as in flies [23]. Nothing has been reported about whether the other five sirtuin proteins (SIRTs 3–7) can influence the survival or death of neurons.

The goal of our study was to gain insight into the roles of different sirtuin proteins in the regulation of neuronal survival. To facilitate a direct comparison of their effects on neuronal viability, we initially utilized a single and well-established paradigm for our study – cultured CGNs. These neurons survive well in medium containing depolarizing levels of potassium but undergo apoptosis in low potassium medium [24]. We report that SIRT1 and SIRT5 protect CGNs from LK-induced apoptosis, while SIRT2, SIRT3 and SIRT6 promote cell death. To investigate whether these effects were also seen in response to other apoptotic stimuli and in other cell types, we subsequently extended our studies to another paradigm of neuronal apoptosis in which HT-22 neuroblastoma cells were induced to die by homocysteic acid treatment [25], [26]. While the effects of a majority of the SIRT proteins were similar to that observed in CGNs, in HT-22 cells SIRT5 was found to be apoptotic while SIRT6 had a mildly protective effect.

## Results

### Effects of sirtuin expression on neuronal survival

**Figure 1:**
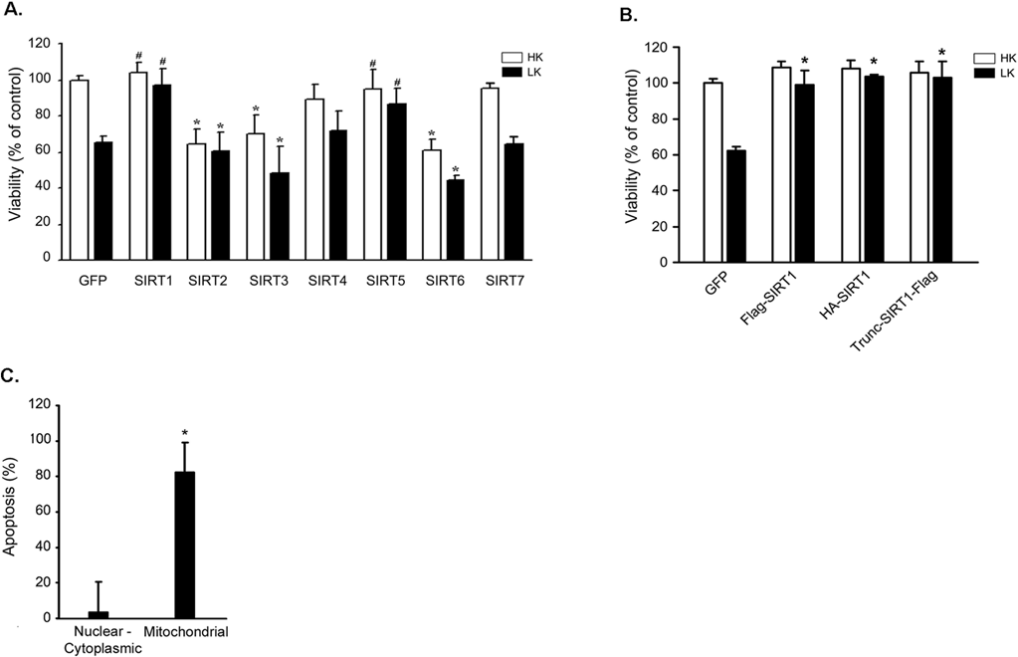
Effect of SIRT 1–7 expression on neuronal survival. (A) Sirtuin family expression and cerebellar granule neuron survival. Expression plasmids encoding Flag-tagged forms of SIRT 1–7 were transfected into CGNs and the cultures were switched to HK or LK medium. The proportion of transfected neurons that were apoptotic was quantified 24 hrs after the switch and compared with control cultures which were transfected with CMV-GFP. Viability for transfected cells was determined using DAPI staining of chromatin (*indicates p<.05 compared with GFP HK (control); # indicates p<.05 compared with GFP LK). (B) Expression of other SIRT1 constructs in neurons confirms neuroprotection. Neurons were transfected with Flag-SIRT1 (tag on the N-terminus), HA-SIRT1, or Trunc-SIRT1-Flag and switched to HK or LK medium. The proportion of transfected neurons that were apoptotic was quantified 24 hrs after the switch and compared with control cultures which were transfected with CMV-GFP. Apoptosis was determined for transfected cells using DAPI staining (* indicates p<.05 compared with GFP LK; control = GFP HK). (C) SIRT5 localization and CGNs survival. SIRT5 localization in CGNs was determined by immunocytochemistry following SIRT5-Flag transfection and viability assessed using DAPI staining 24 after HK and LK treatment. The localization of viable and apoptotic SIRT5-Flag expressing cells was scored as nuclear and cytoplasmic or mitochondrial (* indicates p<.05 compared with nuclear-cytoplasmic).

**Figure 2:**
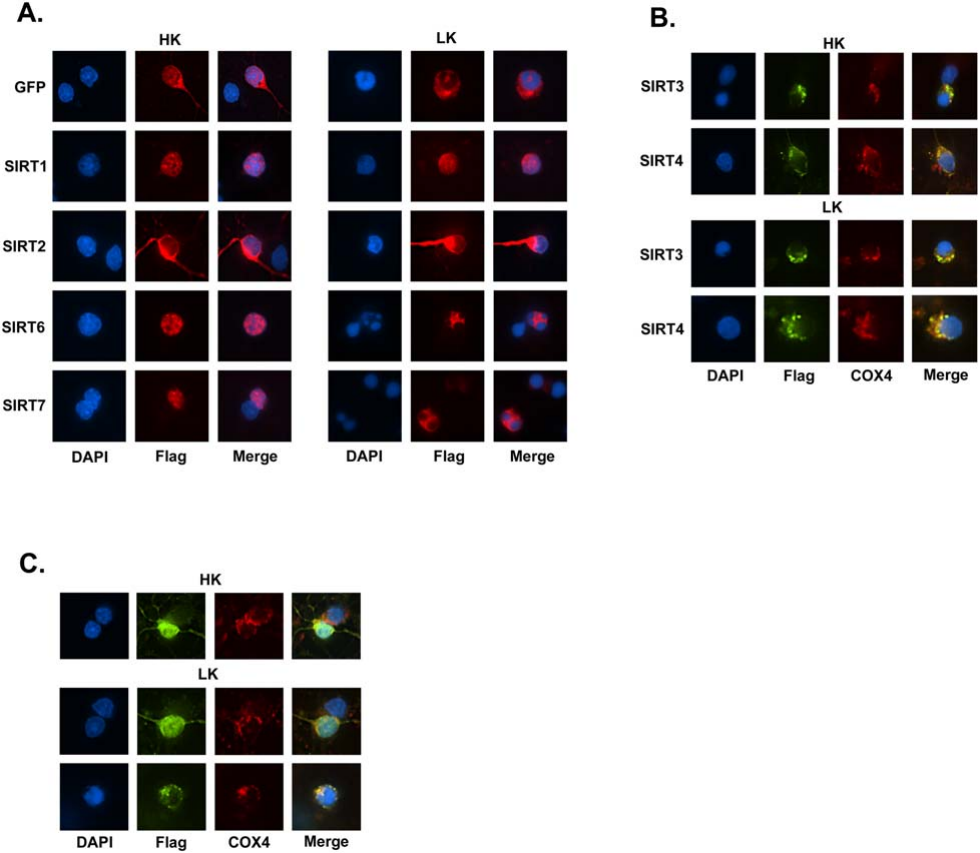
Subcellular localization pattern of SIRT 1–7 in neurons. Expression plasmids encoding Flag-tagged forms of SIRT 1–7 were transfected into CGNs and the cultures were switched to HK or LK medium. The localization of each sirtuin was visualized by immunocytochemistry using a Flag antibody. DAPI staining was used to label the nucleus. In some cases, Cox4 antibody was used to visualize mitochondria. Images were obtained with a Nikon Eclipse 80i using a 60× objective. (A) Subcellular localization of SIRT1, SIRT2, SIRT6, and SIRT7 in HK and LK-treated neuronal cultures. Non-apoptotic cells were used for SIRT2 and SIRT6 in HK to show localization. (B) Mitochondrial localization of SIRT3 and SIRT4. The punctate pattern of SIRT3 and SIRT4 correspond to mitochondrial localization as evidenced by the overlap with Cox4 immunostaining. (C) Subcellular localization of SIRT5 in CGNs. In HK treatment SIRT5 predominately localizes to the cytoplasm, mitochondria, and nucleus (HK panel) while in LK SIRT5 can localize to the cytoplasm, mitochondria, and nucleus (upper LK panel) or to mitochondria (lower LK panel). Mitochondrial localization of SIRT5 is associated with apoptosis.

Cultured CGNs undergo apoptosis when switched from medium containing depolarizing levels of potassium (high potassium or HK medium) to medium containing non-depolarizing levels of potassium (low potassium or LK medium) [24]. We used this tissue culture paradigm to look at the effect of forced expression of each of the seven sirtuin proteins on neuronal survival or death. As shown in Fig. 1A, overexpression of SIRT1 had no effect on neuronal survival in HK, but completely inhibited LK-induced cell death. Although generally present in the nucleus, nucleo-cytoplasmic shuttling of SIRT1 has recently been described [8], [27]. In CGNs, ectopically-expressed SIRT1 localizes to the nucleus in both HK and LK conditions (Fig. 2A). Although the effect of SIRT1 expression has not been previously studied in CGNs, our result is consistent with the neuroprotective action of this sirtuin observed in other cell culture and *in vivo* experimental models of neurodegeneration [14], [20]. Neuroprotection was also observed with SIRT1 conjugated to an N-terminus HA-tag ruling out the possibility that the Flag–tag itself contributed to its protective efficacy (Fig. 1B). We also expressed a truncated version of SIRT1 lacking the first 81 amino acids (Trunc-SIRT1-Flag), but which has been found to retain its catalytic activity [5]. Previous studies have suggested that the N-terminus region of SIRT1 is involved in interaction with other proteins and contains two potential phosphorylation sites (Ser27 and Ser47; [28], [29]). Trunc-SIRT1-Flag was as effective as the full-length form in inhibiting neuronal death (Fig. 1B).

Pharmacological and genetic inhibition of SIRT2 has been shown to protect against α-synuclein toxicity in cultured neurons and in *Drosophila* suggesting that SIRT2 is involved in promoting neurodegeneration [30]. Consistent with these findings, SIRT2 overexpression reduces survival of otherwise healthy neurons (Fig. 1A). In other cell types, SIRT2 is generally cytoplasmic but translocates to the nucleus during mitosis [16]. We observed that SIRT2 displays a cytoplasmic localization in CGNs which is not altered during apoptosis (Fig. 2A).

The overexpression of SIRT3 in CGNs induces death in HK and increases the extent of cell death in LK medium (Fig. 1A). As observed previously, SIRT3 localizes to mitochondria in these neurons (Fig. 2B) [17]. Overexpression of SIRT4 had no effect on neuronal viability in either HK or LK medium (Fig. 1A). Like SIRT3, and as previously reported [17], the localization of SIRT4 in CGNs is mitochondrial (Fig. 2B).

SIRT5 overexpression inhibits LK-induced apoptosis (Fig. 1A). In contrast to previous studies in which SIRT5 expression was restricted to mitochondria of non-neuronal cell types [17], in a majority of CGNs SIRT5 is widely distributed displaying nuclear, cytoplasmic and mitochondrial localization (Fig. 2C). In a small proportion of these neurons, however, SIRT5 localized solely in mitochondria (Fig. 2C). An analysis of the relationship between subcellular localization and neuroprotection reveals that SIRT5 is protective when it localizes to the cytoplasm. In contrast, when localized to the mitochondria, SIRT5 promotes apoptosis (Fig. 1C).

Overexpression of SIRT6 promotes neuronal apoptosis, while SIRT7 has no effect on cell viability in HK or LK medium (Fig 1A). As observed by other investigators in non-neuronal cells, both of these proteins localize predominantly to the nucleus (Fig. 2A).

### Pharmacological modulators of SIRT1 activity have no effect on neuroprotection

**Figure 3:**
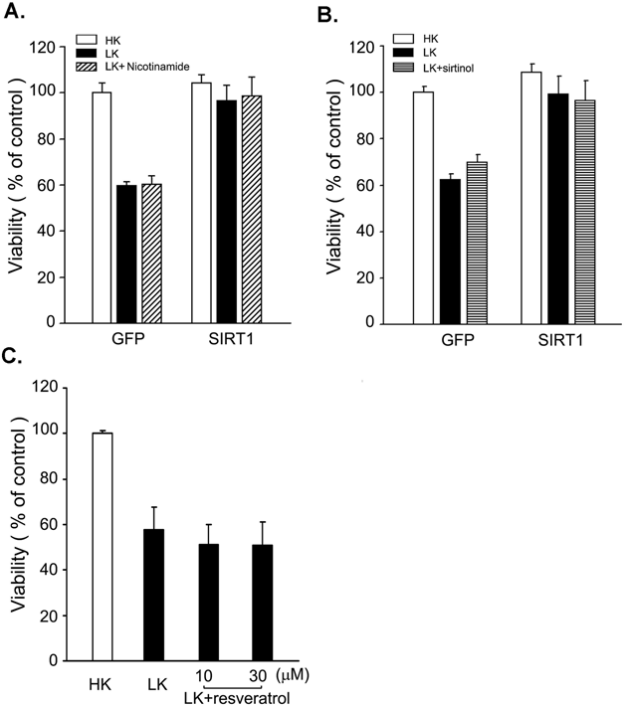
Pharmacological modulators of SIRT1 activity have no effect on neuroprotection. (A) Neurons treated with nicotinamide. CGNs were transfected with Flag-tagged SIRT1 or GFP control vector and the cultures switched to LK medium or LK-medium supplemented with 5 mM nicotinamide. Viability of transfected neurons (detected by Flag immunocytochemistry) was quantified 24 hours later (* indicates p<.05 compared with GFP LK; # indicates p<.05 compared with GFP HK). (B) Sirtinol does not affect SIRT1 protection. Flag-tagged SIRT1 and GFP vectors were transfected into CGNs. The neurons were then treated with LK medium or LK-medium supplemented with 100 µM sirtinol. Survival for SIRT1-transfected neurons was assessed using immunocytochemistry and DAPI staining 24 hours following treatment (* indicates p<.05 compared with GFP LK; # indicates p<.05 compared with GFP HK). (C) Resveratrol cannot rescue neurons in LK. Cultured CGNs were switched to HK medium, LK medium, or LK medium containing 10 or 30 µM resveratrol. Neuronal viability was quantified 24 hours later by DAPI staining.

Several investigators have found that the anti-apoptotic effect of SIRT1 overexpression in neurons as well as non-neuronal cell types is mimicked by chemical agonists of SIRT1 and can be inhibited by pharmacological inhibitors of this sirtuin [11], [31]–[36]. In these paradigms therefore, the effect of SIRT1 is mediated by its enzymatic activity. To examine whether this was also the case in CGNs, we examined whether nicotinamide could block the protective effect of SIRT1 against LK-induced neuronal death. Nicotinamide is an end-product inhibitor that suppresses the activity of all sirtuins by directly binding within a conserved pocket of the enzyme and inhibiting NAD+ hydrolysis [37]–[39]. It is widely used as a sirtuin inhibitor in cell culture studies and nicotinamide's ability to block SIRT1 activity both *in vitro* and in cultured cells has previously been established [5], [10], [37], [40]–[42]. Surprisingly, nicotinamide fails to reduce the neuroprotective activity of SIRT1 in CGNs (Fig. 3A). Another more selective and potent pharmacological inhibitor of SIRT1 is sirtinol [30], [43]–[45]. As seen in Figure 3B, sirtinol had no effect on SIRT1-mediated neuronal survival. We also used splitomicin, a recently developed sirtuin inhibitor [46], [47]. Like nicotinamide and sirtinol, treatment of neurons with splitomicin failed to block neuroprotection by SIRT1 in LK (data not shown).

Resveratrol is a potent pharmacological agonist of SIRT1 [48]. Treatment with this polyphenol induces deacetylation of known SIRT1 substrates such as NF-κB, PGC-1α, and p53, and can mimic the anti-apoptotic and anti-tumor activity of SIRT1 [21], [48]–[51]. It has been shown that resveratrol activates SIRT1 and recapitulates its biological effects in cultured cells at 10 uM [52]–[56]. As shown in Fig. 3C and as previously reported by Alvira et al. (2007), treatment with resveratrol at doses of up to 30 µM did not prevent LK-mediated death of neurons. Our results from pharmacological activation and inhibition of SIRT1 suggest that SIRT1-mediated neuroprotection is independent of its deacetylase activity in CGNs.

### Mutant forms of SIRT1 lacking deacetylase activity are completely neuroprotective

**Figure 4:**
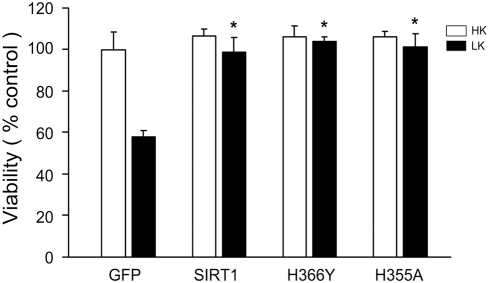
A deacetylase-deficient form of SIRT1 is neuroprotective. CGNs were transfected with vectors encoding GFP, Flag-tagged wild-type form of SIRT1, or the deacetylase-dead mutation forms of SIRT1, H363Y or H355A. The cultures were then switched to HK or LK medium for 24 hours and viability of transfected neurons was quantified by DAPI staining (* indicates p<.05 compared with GFP LK).

To directly test the possibility that neuroprotection by SIRT1 was not dependent on its catalytic activity, we used a point-mutant form of SIRT1-H363Y which is catalytically inactive [14], [40], [57]. Figure 4 shows that this catalytically-inactive form of SIRT1 was as protective as the wild-type form when overexpressed in neurons. Complete neuroprotection was also observed with SIRT1-H355A, another catalytically-dead mutant form of SIRT1 that is commonly used (Fig. 4) [9], [58]–[60]. Taken together with the failure of SIRT1 inhibitors to reduce neuroprotection, this result confirms that the deacetylase activity of SIRT1 is not required for neuroprotection in CGNs.

#### Forced sirtuin expression in the HT-22 mouse neuroblastoma cell line

**Figure 5:**
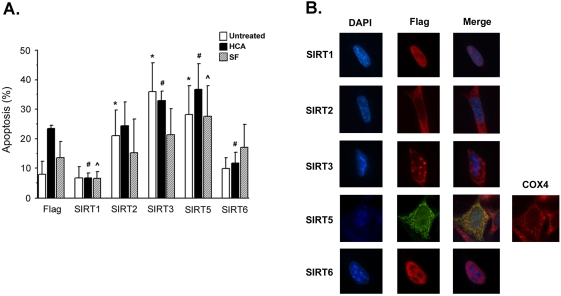
Forced sirtuin expression in the HT-22 cells. (A) Effect of sirtuin expression on HT-22 cell survival. Sirtuin expression levels were enhanced in HT-22 neuroblastoma cells via transfection with mammalian expression plasmids. HT-22 cells were treated 24 hours following transfection with normal growth medium (untreated), 1 mM HCA or Serum-free (SF) medium for 24 hours. The viability of transfected cells was determined using a TUNEL assay. The mean of 5 experiments were taken (* indicates p<.05 compared with Flag untreated sample; # indicates p<.05 compared with Flag HCA sample; ˆ indicates p<.05 compared with Flag SF sample). (B) Subcellular localization of exogenous SIRT1, SIRT2, SIRT3, SIRT5 and SIRT6 in HT-22 cells. HT-22 cells were transfected with SIRT-Flag constructs and immunocytochemistry performed using a Flag antibody. DAPI staining was used to visualize nuclei/chromatin. SIRT5 shows colocalization with the mitochondrial protein Cox4.

We next examined whether the effects observed with SIRT1, SIRT2, SIRT3, SIRT5, and SIRT6 on the regulation of LK-induced neuronal death extended to mouse HT-22 cells, a hippocampally-derived neuroblastoma cell line. HT-22 cells grow robustly in serum-containing medium but undergo apoptosis when maintained in serum-free medium. Apoptosis is also induced in these cells following treatment with homocysteic acid (HCA), a glutathione-depleting drug that causes oxidative stress [25], [26], [61]. We examined the effect of expressing each of the five SIRT proteins in HT-22 cells maintained in normal serum medium, treated with serum-free medium or treated with HCA. As shown in Fig. 5A, SIRT1 protects against HCA-induced and serum withdraw-induced apoptosis indicating that its protective action is versatile. Recent reports have described that SIRT1 shuttles to the cytoplasm upon oxidative stress rendering it incapable of exerting its inactivating effect on anti-apoptotic substrates within the nucleus and consequently sensitizing cells to oxidative-stress mediated apoptosis [8], [27]. In HT-22 neuroblastoma cells however, SIRT1 is in the nucleus even following HCA-induced oxidative stress (Fig. 5B). As observed in CGNs, SIRT2 overexpression had an apoptotic effect on untreated neuroblastoma cells. However, SIRT2 did not enhance serum deprivation or HCA-induced cell death (Fig. 5A). As observed in neurons and in other cell types, SIRT2 was observed exclusively in the cytoplasm of HT-22 cells (Fig. 5B). In contrast to its generally protective action in CGNs, SIRT5 expression induces apoptosis in otherwise healthy neuroblastoma cells and also exacerbates the toxic effect of HCA. Also in contrast to CGNs in which SIRT5 was generally not restricted to the mitochondria, we find that SIRT5 colocalizes with the mitochondrial marker Cox4 in HT-22 cells (Fig. 5B). Such a mitochondrial pattern of localization has been described for SIRT5 previously [17]. Expression of SIRT3 promotes apoptosis in neuroblastoma cells and enhances HCA lethality, whereas SIRT6 does not induce HT-22 cell death (Fig. 5A). In fact, SIRT6 exhibits a modest protective effect against HCA treatment contrary to its toxic role in the CGN apoptosis paradigm used in this study. Both SIRT3 and SIRT6 exhibit localization patterns similar to those seen in CGNs and consistent with previous reports in other cell types (Fig. 5B) [17].

## Discussion

Emerging evidence suggests that sirtuins are involved in the regulation of neuronal survival and death. The overall consensus from these studies is that SIRT1 has neuroprotective activity that is mediated by deacetylation of molecules such as p53 and PGC-1α [10], [22]. In contrast, SIRT2 has been reported to promote neurodegeneration in experimental models of Parkinson's disease [23]. In this study we examined the influence of all seven members of this sub-family of HDAC proteins on the survival of CGNs. It is noteworthy that the activities of SIRTs 3–7 have not been previously studied in the context of neuronal survival. We report for the first time that SIRT5 has neuroprotective activity, while SIRT3 and SIRT6 are neurotoxic when overexpressed in neurons. With the exception of SIRT5, the subcellular localization pattern of these sirtuin proteins in CGNs is as previously reported in non-neuronal cell types. Whereas SIRT5 is strictly mitochondrial in other cells types, in CGNs it is generally present in the cytoplasm, nucleus and mitochondria with only a small proportion of cells displaying selective mitochondrial localization. We find that when localized to the mitochondria the protective activity of SIRT5 is replaced by a pro-apoptotic effect. More work is necessary to delineate what factors and mechanisms determine the localization of SIRT5 within cells.

To test whether the various sirtuins found to affect survival of CGNs have similar effects in another model of neuronal apoptosis, we used hippocampus-derived HT-22 neuroblastoma cells. While the anti- and pro-survival effects of SIRT1, SIRT2, and SIRT3 were preserved in HT-22 cells, there were differences in the cases of SIRT5 and SIRT6. Expression of SIRT5, which is generally protective in CGNs, is apoptotic in HT22 cells and potentiates the level of cell death induced by HCA treatment or serum withdrawal. In CGNs protection by SIRT5 is associated with a non-mitochondrial subcellular localization. In fact, neuronal viability is low when this protein is localized to mitochondria. In HT-22 neuroblastoma cells, SIRT5 is almost always mitochondrially-associated (Fig. 5B). These observations suggest that the opposing action of SIRT5 on neuronal viability is likely to be dictated by its subcellular localization. In the case of SIRT6, the pro-apoptotic action seen in CGNs is not observed in HT-22 cells although its subcellular localization pattern is the same in both cell types. This result suggests that differences in intracellular context or protein-protein interactions may determine activity of SIRT6 with respect to the regulation of apoptosis.

All biological effects of sirtuins have so far been attributed to their enzymatic activity. Intensive effort is being spent on identifying small-molecule pharmacological modulators of sirtuin activity as therapeutic tools to treat neurodegenerative diseases as well as other age-related disorders such as type 2 diabetes, cancer, inflammation and cardiovascular disease (reviewed in [1]; [3], [4], [21]). An interesting and novel finding of our study is that the neuroprotection by SIRT1 is not affected by nicotinamide, a potent and noncompetitive inhibitor of the sirtuins. Sirtinol, a pharmacological inhibitor of SIRT1 with a distinct mechanism of action, also fails to reduce neuroprotection by SIRT1. Other investigators have established the selectivity and potency of sirtinol as a selective SIRT1 inhibitor by showing that its biological effects are faithfully mimicked by SIRT1 siRNA and that it can neutralize the actions of pharmacological SIRT1 activators [22]. We also find that resveratrol, a commonly-used agonist of SIRT1 as well as other sirtuins, does not protect neurons against LK-induced neuronal death. This finding is consistent with the results of Alvira et al., 2007 who also failed to observe protection with resveratrol in LK-treated CGNs [35]. Most convincingly, we find that two separate point-mutant forms of SIRT1, H363Y and H355A, are both as protective as wild-type SIRT1 in CGNs. Previous work by a number of other laboratories has established that both of these two mutant forms of SIRT1 have no detectable deacetylase activity *in vitro* or in transfected cells [9], [14], [57]–[60], [62]. SIRT1 also has weak ADP-ribosyltransferase activity [63]. This ADP-ribosyltransferase activity is required for the interaction of SIRT1 with PGC1α, but not its interaction with with p53 or Foxo3a [42]. We have used a mutant SIRT construct, SIRT1-G261A which lacks ADP-ribosyltransferase activity [42] and found that its overexpression is fully protective against LK-induced neuronal death (Ma and D'Mello, unpublished observation). Our results therefore suggest a novel, non-catalytic mechanism of action for SIRT1-mediated neuroprotection. Further effort will be necessary to define what this mechanism is but one possibility is that it involves interactions between SIRT1 and other apoptosis-regulatory proteins. Association between SIRT1 and p53, p65, c-jun and E2F1, all of which are involved in regulating neuronal apoptosis, has previously been reported (reviewed in [1]; [55], [64]–[66]).

An obvious caveat of our study is that it relies solely on ectopically-expressed proteins. It is likely that overexpression represents an amplification of the normal activity of these proteins on neuronal survival. But it is also possible that the effects we observe represent an artifact of overexpression and as such have little physiological relevance. Further investigation using siRNA-mediated suppression of endogenous sirtuins or utilization of cultures from knock-out mice lacking individual sirtuins will be necessary to study this issue. Besides physiological relevance however, our finding that SIRT1 and SIRT5 are neuroprotective when expressed at elevated levels raises the possibility that increasing endogenous expression of these two proteins within vulnerable neuronal populations pharmacologically, or ectopically expressing them at high levels (using viral vectors for example) could represent viable therapeutic approaches to prevent neuronal loss in neurodegenerative diseases.

In conclusion, our study represents the first investigation of the effects of SIRTs 3–7 on the regulation of neuronal survival. Moreover, we show for the first time that in addition to inhibiting cell death by deacetylating apoptosis-regulatory proteins, SIRT1 can act through a novel, catalytic-independent mechanism to protect against apoptosis. And finally, we show that SIRT5 can have contrasting effects on neuronal viability, which is dictated by its subcellular localization.

## Materials and Methods

### Materials

Unless otherwise indicated, all tissue culture reagents and Lipofectamine 2000 were purchased from Invitrogen (Invitrogen, Carlsbad, CA). Flag antibody, cytosine arabinofuranoside and DAPI were from Sigma (St. Louis, MO). Cox4 antibody was from Santa Cruz Biotechnology(Santa Cruz, CA). Expression plasmids for Flag-tagged SIRT1–SIRT7, HA-tagged SIRT1, Flag-tagged SIRT1-H363Y and Flag-tagged SIRT-G261A were purchased from Addgene (Addgene, Cambridge, MA). Flag-tagged SIRT1-H355A was provided by Dr. Jiandong Chen (H. Lee Moffitt Cancer Center, Tampa, FL). All cDNAs were sequenced and the expression of a protein of the correct size verified following transfection of HEK293T cells.

### Culturing of cerebellar granule neurons and HT-22 cells

Cerebellar granule neurons were cultured from 7–8 day old Wistar rats (Charles River, Wilmington, MA) as previously described [24]. The cultures were plated in 24-well poly-L-lysine coated dishes with glass coverslips at a density of 1×10^6^ cells/well in basal Eagle's medium with Earle's salts (BME) supplemented with 10% FBS, 25 mM KCl, 2 mM glutamine and 100 µg/mL gentamicin. To prevent the proliferation of the small number of non-neuronal cells, cytosine arabinofuranoside (10 µM) was added to the culture medium about 20 hrs after plating. HT-22 cells were maintained in Dulbecco's Modified Eagle's Medium (DMEM) supplemented with 10% FBS, penicillin and streptomycin.

### Transfection of primary neurons

The neuronal cultures were transfected 4–5 days after plating using cesium chloride-purified plasmid DNA and the calcium phosphate method as previously described [67]. 3 µg/mL of plasmid DNA was used per well. A day after transfection, the cells were rinsed once and then maintained in serum-free BME medium containing either 5 mM KCl (low potassium, LK) or 25 mM KCl (high potassium, HK). Control cultures were transfected with CMV-GFP. Transfected neurons were detected by GFP fluorescence or by immunostaining using Flag antibody (for all seven sirtuin proteins). Neuronal viability was quantified by staining cell nuclei with DAPI as previously described [67], [68]. Condensed or fragmented nuclei were scored as apoptotic. Double staining was performed to determine the subcellular localization of mitochondrial SIRTs. Ectopically expressed SIRTs were detected using a Flag antibody and a Cox4 antibody was used to label mitochondria. For immunocytochemistry, both Flag antibody and Cox4 antibody were used at 1∶200 dilution.

### Transfection of HT-22 cells

Cells were plated at a density of 1.5×10^4^ cells/well in 24 well plates in antibiotic-free DMEM medium with 10% FBS. Transfection was carried out using 1 µg/mL of plasmid DNA and Lipofectamine 2000 according to manufacturer's instructions. Exogenous genes were allowed to express for 24 hours at which time the cells were treated with fresh normal growth medium, 1 mM HCA or DMEM without FBS. Approximately 17 hours after treatment the viability of transfected cells was determined using immunocytochemistry and a TUNEL assay (Promega, Madison, WI) that detects cleaved DNA characteristic of apoptotic cells.

### Statistics

Graphs represent mean values obtained from three or more independent experiments. Error bars show standard deviation from three separate experiments. Statistical analysis for graphical data was performed using an unpaired t-test (student t-test). P values of <0.05 were deemed statistically significant.
